# Clinical outcomes of residual branch vessel dissection following type A aortic dissection repair

**DOI:** 10.3389/fmed.2026.1913510

**Published:** 2026-08-26

**Authors:** Jing Li, Siyuan Chen, Changying Zhao, Yang Yan

**Affiliations:** Department of Cardiovascular Surgery, First Affiliated Hospital of Xi’an Jiaotong University, Xi’an, China

**Keywords:** aortic arch replacement, long-term prognosis, residual dissection in the arch branch vessels, type A aortic dissection, win ratio analysis

## Abstract

**Objectives:**

Although aortic arch replacement eliminates the primary entry tear in type A aortic dissection, residual dissection in arch branch vessels (RD-ABV), often linked to fragile distal tissue or residual tears, may sustain false lumen patency and be related to post-discharge recovery. The clinical relevance of RD-ABV remains unclear.

**Methods:**

This retrospective cohort study included patients who underwent total arch replacement for aortic dissection between January 2018 and December 2024. Patients with in-hospital postoperative cerebral infarction, cerebral hemorrhage, or death were excluded, and follow-up began on the date of discharge. RD-ABV was assessed using the first postoperative CTA before discharge. The primary outcome was a composite event including modified Rankin Scale (mRS) 3–6, cerebral infarction (CI), cerebral hemorrhage (CH), and cardiovascular and cerebrovascular reoperation. Secondary outcomes included CI, CH, and reoperation. IPTW-adjusted win ratio was used for analyses of the primary and secondary composite outcomes.

**Results:**

A total of 564 patients were included, of whom 156 had RD-ABV. The IPTW-adjusted win ratio of the primary composite outcome was 0.47 (95% CI: 0.27–0.82; *P* = 0.007), whereas the secondary composite outcome did not reach statistical significance (win ratio, 0.56; 95% CI: 0.30–1.08; *P* = 0.082). In IPTW-weighted Kaplan-Meier sensitivity analyses, lower event-free estimates were observed for the primary outcome (raw event-free counts, 123/156 [78.8%] vs. 355/408 [87.0%]; weighted log-rank *P* = 0.014), mRS 3–6 (133/156 [85.3%] vs. 387/408 [94.9%]; weighted log-rank *P* < 0.001), and CH (151/156 [96.8%] vs. 406/408 [99.5%]; weighted log-rank *P* = 0.034). No statistically significant differences were observed for the secondary composite outcome, CI, or reoperation. Hierarchical analyses indicated that the primary composite outcome was mainly driven by mRS 3–6.

**Conclusion:**

Among selected post-discharge survivors after aortic arch replacement, RD-ABV was associated with worse mid-term functional recovery, mainly reflected by mRS 3–6. The secondary composite outcome excluding mRS did not reach statistical significance. These findings should be interpreted as hypothesis-generating and require prospective validation.

## 1 Introduction

Aortic dissection is a life-threatening cardiovascular emergency characterized by a tear in the intimal layer of the aorta, allowing blood to enter the medial layer and create a false lumen (1). This pathological process can result in severe complications, including cardiac tamponade, coronary artery involvement, and cerebral ischemia (2). Importantly, the dissection flap can extend into and acutely compromise branch vessels originating from the aortic arch.

Surgical management of dissections involving the aortic root, ascending aorta, or aortic arch typically includes replacement of the affected segments with synthetic grafts, often incorporating the proximal portions of arch branch vessels (3). Nevertheless, despite successful repair of the primary aortic tear, residual dissection extending into the distal portions of these branches can persist postoperatively (4), potentially compromising long-term prognosis (5, 6).

Studies have suggested that residual dissection of the brachiocephalic artery is associated with increased risk of ipsilateral ischemic events, particularly cerebral ischemia (5). Additionally, aortic dilation and residual dissection have both been associated with adverse prognosis (7), suggesting that their coexistence may be linked to a less favorable disease course and poorer long-term outcomes (8–11). Distal aortic expansion, in particular, has been reported to be associated with late death (8, 10, 11). Furthermore, some studies have indicated that persistent false lumen flow related to distal residual dissection may contribute to continuous aortic expansion and adverse remodeling of the distal vessels (12, 13). This phenomenon suggests that residual dissection may be associated with poorer prognosis. However, studies have indicated that although complete absence of false lumen thrombosis is associated with more rapid aortic expansion, it carries a significantly lower risk of early mortality compared to partial thrombosis (14).

The hemodynamic consequences of persistent false lumen flow and its relationship with end-organ perfusion, especially cerebral blood flow, remain incompletely understood. Altered flow dynamics and sustained mural stress in dissected branch arteries may be associated with chronic malperfusion syndromes, aneurysmal degeneration, or late neurologic events, which are critical areas for further investigation. In addition, the potential role of advanced imaging modalities and secondary interventions, including endovascular stenting or extended arch reconstruction, requires further prospective evaluation.

Given these uncertainties, further research is warranted to clarify the clinical implications of branch vessel involvement in aortic dissection and its association with patient outcomes, particularly in areas such as post-discharge recovery and cerebral perfusion abnormalities. Elucidating the natural history and hemodynamic significance of residual branch dissections may help identify patients who require closer surveillance and may inform the design of future interventional studies.

## 2 Patients and methods

### 2.1 Study design and enrollment

**FIGURE 1:**
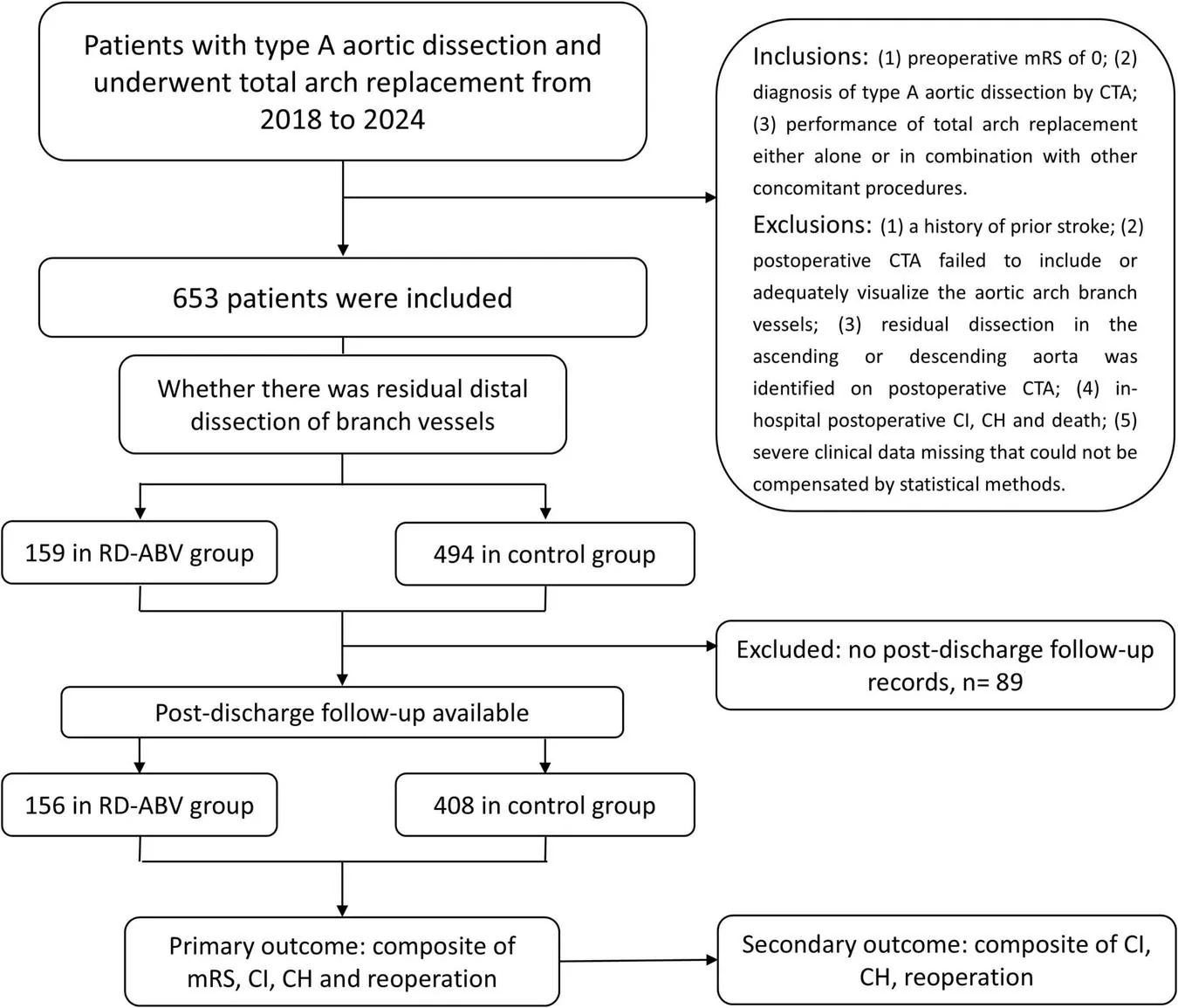
Flowchart of patient screening, exclusion, follow-up availability, grouping, and outcomes in this study.

This retrospective cohort study screened 1,351 patients with aortic dissection treated at the First Affiliated Hospital of Xi’an Jiaotong University from 2018 to 2024. The inclusion criteria were as follows: (1) preoperative modified Rankin Scale (mRS) score of 0; (2) diagnosis of type A aortic dissection by computed tomography angiography (CTA); (3) performance of total arch replacement either alone or in combination with other concomitant procedures. The exclusion criteria were as follows: (1) a history of prior stroke; (2) postoperative CTA failed to include or adequately visualize the aortic arch branch vessels; (3) residual dissection in the ascending or descending aorta was identified on postoperative CTA; (4) in-hospital postoperative cerebral infarction (CI), cerebral hemorrhage (CH), or death; (5) missing data accounted for more than 50% of the total variables. After applying these inclusion and exclusion criteria, 653 patients remained eligible. Patients with completely unavailable post-discharge follow-up information were further excluded (*n* = 89), leaving 564 patients in the final analytic cohort. Therefore, this study cohort represents selected post-discharge survivors without early major neurological events and with available post-discharge outcome information. The flowchart of this study is shown in Figure 1.

All cases in this study were from the CLASSIC cohort, registered at ClinicalTrials.gov (NCT05999188) with ethics approval (No. XJTU1AF2023LSK-313). This study protocol was approved by the institutional ethics committee (No. XJTU1A2025LSYY-350) under the Declaration of Helsinki.

### 2.2 Grouping

Enrolled patients were divided into residual dissection in arch branch vessels (RD-ABV) group and non-RD-ABV group based on the presence of residual distal dissection in the brachiocephalic trunk, left common carotid artery, or left subclavian artery on the first postoperative CTA examination performed after surgery and before discharge (5, 15, 16). For patients with RD-ABV, the specific involved branch vessel and the number of dissected arch branches were recorded for descriptive reporting. The routine postoperative CTA protocol covered the coronary arteries and the thoracic and abdominal aorta, allowing assessment of the aortic arch and proximal arch branch vessels rather than a dedicated head and neck CTA evaluation. CTA images were reviewed independently by two qualified physicians, and interobserver consistency was compared. Discrepant assessments were adjudicated by a third imaging expert. Detailed morphological features, including false lumen patency, degree of true lumen narrowing, thrombosis status, distal extension into the carotid or vertebral arteries, and imaging signs of malperfusion, were not systematically annotated during the original retrospective imaging review and therefore were unavailable for further analysis.

### 2.3 Data collection and study outcomes

The baseline characteristics included demographic information, comorbidities (such as hypertension, coronary artery disease, valvular disease, and diabetes), and biochemical test results at admission. To enhance the clarity and relevance of the baseline characteristics table, we prioritized biochemical markers that are clinically related to vascular and systemic inflammatory responses and have been associated with disease progression and prognosis, such as neutrophil count, platelet count, D-dimer, creatinine, aspartate aminotransferase, and alanine aminotransferase (17, 18). In addition, we considered other clinically relevant factors that have been associated with prognosis (19). Follow-up started on the date of discharge. Follow-up information was mainly collected via outpatient visits and telephone calls to patients or their relatives. The mRS assessment was preferentially obtained from medical records. When the mRS score was not documented in the medical record, trained medically qualified assessors conducted structured telephone follow-up with patients or their relatives. All assessors received unified training and were blinded to RD-ABV grouping.

The primary outcome was a composite outcome that included mRS 3–6, cerebral infarction (CI), cerebral hemorrhage (CH), and cardiovascular and cerebrovascular reoperation (20). Post-discharge all-cause death was recorded as an all-cause mortality event during follow-up and classified as mRS 6 within the mRS outcome. In-hospital deaths were excluded according to the prespecified exclusion criteria. The secondary outcome was a composite outcome including CI, CH, and cardiovascular and cerebrovascular reoperation, excluding mRS.

### 2.4 Statistical analysis

Variables with missing values exceeding 20% were excluded from the analysis; these were predominantly intraoperative and procedural variables, including cardiopulmonary bypass time, aortic cross-clamp time, circulatory arrest time, cerebral perfusion strategy, cerebral perfusion duration, cannulation strategy, frozen elephant trunk use, branch reconstruction technique, concomitant surgical procedures, intraoperative blood loss, blood transfusion volume, and surgeon-specific information. For the remaining variables, multiple imputation by chained equations was performed to generate ten imputed datasets, with results reported based on the pooled estimates. Continuous variables were expressed as mean ± standard deviation (SD) or median with interquartile range (IQR), as appropriate, while categorical variables were presented as counts and percentages. The median follow-up time was estimated using the reverse Kaplan-Meier method.

To reduce measured confounding and control for baseline differences, we performed inverse probability of treatment weighting (IPTW) using a propensity score derived from a logistic regression model, which incorporated observed baseline covariates. Following IPTW adjustment, baseline balance was evaluated using standardized mean differences (SMD), with an SMD < 0.1 indicating optimal balance between the RD-ABV and non-RD-ABV groups. Because WBC, neutrophil count, and lymphocyte count were biologically and statistically correlated, an additional sensitivity propensity score model excluding WBC while retaining neutrophil and lymphocyte counts was performed to assess the robustness of weight distribution, covariate balance, and outcome estimates (Supplementary Table 9).

The primary analysis was conducted in the IPTW-weighted cohort using the Win Ratio (WR) method. Every patient in the RD-ABV group was compared with every patient in the non-RD-ABV group during a shared follow-up time, defined as the minimum of their respective follow-up times. Matched pairs were compared hierarchically based on a pre-specified priority sequence of time-to-event outcomes: time to mRS 3–6, time to CI, time to CH, and time to reoperation. Within each hierarchical layer, a patient was defined as the “winner” if they remained event-free or experienced the event later than their counterpart. Pairs remaining tied after all layers were classified as “tied” in the overall calculation. In this study, a Win Ratio (WR) < 1.0 was interpreted as indicating a poorer prognosis for the RD-ABV group compared to the non-RD-ABV group. Furthermore, dynamic changes in the IPTW-adjusted Win Ratio over the follow-up period were evaluated to assess the temporal stability of the clinical outcomes. To further evaluate the robustness of our primary findings, sensitivity analyses were conducted in the IPTW-weighted cohort. Specifically, event-free survival probabilities for both the composite outcomes and their individual components were estimated using the Kaplan-Meier method, with differences between the groups compared using the IPTW-weighted log-rank test. For IPTW-weighted Kaplan-Meier analyses, event counts were reported as raw numbers for descriptive purposes, whereas survival probabilities and P values were estimated in the IPTW-weighted cohort. Furthermore, multivariable Cox proportional hazards regression models were constructed to examine the association between RD-ABV and these outcomes after adjustment for measured covariates. Variables demonstrating a *P*-value < 0.10 in univariable screening, together with selected clinically relevant baseline covariates, were incorporated into the multivariable models. The results of the Cox regression analyses were reported as hazard ratios (HRs) and 95% confidence intervals (CIs). All statistical analyses were performed using R version 4.5.1, and a two-sided *p*-value < 0.05 was considered statistically significant.

## 3 Results

### 3.1 Characteristics of the participants

Of the 1,351 patients screened between 2018 and 2024, 653 remained eligible after applying the inclusion and exclusion criteria. After excluding 89 patients with completely unavailable post-discharge follow-up information, 564 patients were included in the final analysis; 156 (27.7%) were assigned to the RD-ABV group and 408 (72.3%) to the non-RD-ABV group. Among patients with RD-ABV, the brachiocephalic trunk was involved in 105 (67.3%), the left common carotid artery in 46 (29.5%), and the left subclavian artery in 45 (28.8%). Single-, two-, and three-branch involvement was observed in 124 (79.5%), 24 (15.4%), and 8 (5.1%) patients, respectively (Supplementary Table 8). Because subgroup sizes and outcome event counts were limited, no formal outcome analysis by anatomical involvement pattern was performed. Baseline characteristics before and after IPTW are presented in Table 1. Before IPTW, D-dimer levels were higher in the RD-ABV group than in the non-RD-ABV group [10.25 (4.44, 23.49) vs. 7.10 (2.65, 17.93); *P* = 0.002]. In contrast, the proportion of hypertension [94 (60.3%) vs. 299 (73.3%); *P* = 0.003], lymphocyte count [0.74 (0.49, 1.06) vs. 0.84 (0.59, 1.21); *P* = 0.006], direct bilirubin [3.90 (2.40, 6.20) vs. 4.40 (2.80, 6.80); *P* = 0.048], and alanine aminotransferase [22.00 (17.00, 30.00) vs. 25.00 (19.00, 37.00); *P* = 0.002] were lower in the RD-ABV group. After IPTW, most measured baseline covariates were balanced, although residual imbalance remained for total bilirubin and direct bilirubin. The sum of stabilized weights was 158.9 in the RD-ABV group and 407.1 in the non-RD-ABV group, with an overall effective sample size of 497.8. Detailed information on the propensity score model, stabilized weight distribution, common support, effective sample size, and covariate balance diagnostics is provided in Supplementary Table 4–7 and Supplementary Figures 4–6. Detailed information on post-discharge medications for the two groups was presented in Supplementary Table 1.

**TABLE 1:** Baseline characteristics of the two groups.

Characteristics	Before IPTW			After IPTW		
	RD-ABV group (*n* = 156)	Non-RD-ABV group (*n* = 408)	| SMD|	RD-ABV group (weighted *n* = 158.9)	Non-RD-ABV group (weighted *n* = 407.1)	| SMD|
Gender-male (*n*, %)	121 (77.6)	307 (75.2)	0.055	117.1 (73.7)	308.5 (75.8)	0.048
Age (years)	53.50 (46.75, 61.00)	54.00 (44.00, 61.00)	0.007	53.07 ± 11.22	52.74 ± 11.42	0.029
Smoking (*n*, %)	84 (53.8)	213 (52.2)	0.033	79.8 (50.2)	212.9 (52.3)	0.042
Alcohol use (*n*, %)	37 (23.7)	104 (25.5)	0.041	36.1 (22.7)	100.6 (24.7)	0.047
Hypertension	94 (60.3)	299 (73.3)	0.279	113.0 (71.1)	283.7 (69.7)	0.031
CHD	18 (11.5)	41 (10.0)	0.048	15.5 (9.8)	41.8 (10.3)	0.017
VHD	6 (3.8)	13 (3.2)	0.036	6.7 (4.2)	14.7 (3.6)	0.03
Diabetes	5 (3.2)	8 (2.0)	0.079	3.9 (2.4)	9.6 (2.4)	0.004
Biochemical factors						
RBC (10^12/L)	4.17 (3.78, 4.55)	4.26 (3.87, 4.66)	0.168	4.20 ± 0.54	4.23 ± 0.63	0.052
WBC (10^9/L)	11.05 (8.95, 13.54)	10.84 (8.82, 14.06)	0.032	11.47 ± 3.62	11.51 ± 4.12	0.012
NEU (10^9/L)	9.66 (7.70, 12.20)	9.30 (7.28, 12.49)	0.008	9.95 ± 3.56	10.00 ± 3.99	0.014
LYMPH (10^9/L)	0.74 (0.49, 1.06)	0.84 (0.59, 1.21)	0.279	0.91 ± 0.49	0.92 ± 0.48	0.019
Platelet (10^9/L)	153.50 (119.75, 193.25)	157.50 (124.00, 200.00)	0.087	166.29 ± 63.40	166.51 ± 65.82	0.003
D-dimer (mg/L)	10.25 (4.44, 23.49)	7.10 (2.65, 17.93)	0.127	16.63 ± 23.30	17.60 ± 29.18	0.037
Fibrinogen (g/L)	2.55 (1.97, 3.18)	2.66 (2.12, 3.42)	0.087	3.04 ± 1.58	2.95 ± 1.35	0.064
TT (s)	17.75 (16.67, 19.40)	17.70 (16.70, 18.90)	0.015	17.50 (16.60, 19.20)	17.70 (16.70, 19.00)	0.038
APTT (s)	29.87 (28.53, 32.60)	30.03 (28.31, 32.09)	0.028	30.95 ± 4.56	30.96 ± 6.17	0.001
PT (s)	14.20 (13.50, 15.00)	14.00 (13.30, 14.90)	0.041	14.22 ± 1.58	14.39 ± 3.48	0.065
INR	1.11 (1.05, 1.19)	1.10 (1.04, 1.18)	0.008	1.15 ± 0.27	1.16 ± 0.43	0.033
Cr (umol/L)	75.50 (55.00, 106.00)	72.00 (57.00, 96.25)	0.019	87.69 ± 51.55	87.37 ± 69.62	0.005
BUN (mmol/L)	6.28 (5.26, 7.87)	6.25 (5.07, 7.69)	0.081	6.87 ± 2.78	6.77 ± 2.66	0.039
Albumin (g/L)	39.10 (35.68, 43.02)	39.45 (35.90, 43.50)	0.024	42.73 ± 13.48	42.85 ± 13.57	0.009
TBIL (umol/L)	18.00 (12.67, 24.33)	18.00 (12.80, 26.42)	0.11	22.93 ± 14.49	20.90 ± 12.27	0.151
DBIL (umol/L)	3.90 (2.40, 6.20)	4.40 (2.80, 6.80)	0.065	6.65 ± 8.41	5.47 ± 4.49	0.176
AST (U/L)	23.50 (20.00, 32.00)	24.00 (19.23, 35.25)	0.152	47.75 ± 76.61	43.96 ± 90.99	0.045
ALT (U/L)	22.00 (17.00, 30.00)	25.00 (19.00, 37.00)	0.164	36.42 ± 48.23	35.59 ± 50.90	0.017

IPTW, inverse probability of treatment weighting; RD-ABV, residual dissection in the arch branch vessels; SMD, standardized mean difference; CHD, coronary heart disease; VHD, valvular heart disease; RBC, red blood cell; WBC, white blood cell; NEU, neutrophil count; LYMPH, lymphocyte count; TT, thrombin time; APTT, activated partial thromboplastin time; PT, prothrombin time; Cr, creatinine; BUN, blood urea nitrogen; INR, international normalized ratio; TBIL, total bilirubin; DBIL, direct bilirubin; AST, aspartate aminotransferase; ALT, alanine aminotransferase. For TT, post-IPTW values are presented as weighted median (IQR) because of the skewed distribution and extreme values; the SMD was calculated using weighted means and variances.

### 3.2 Win ratio analysis of the primary outcomes

**FIGURE 2:**
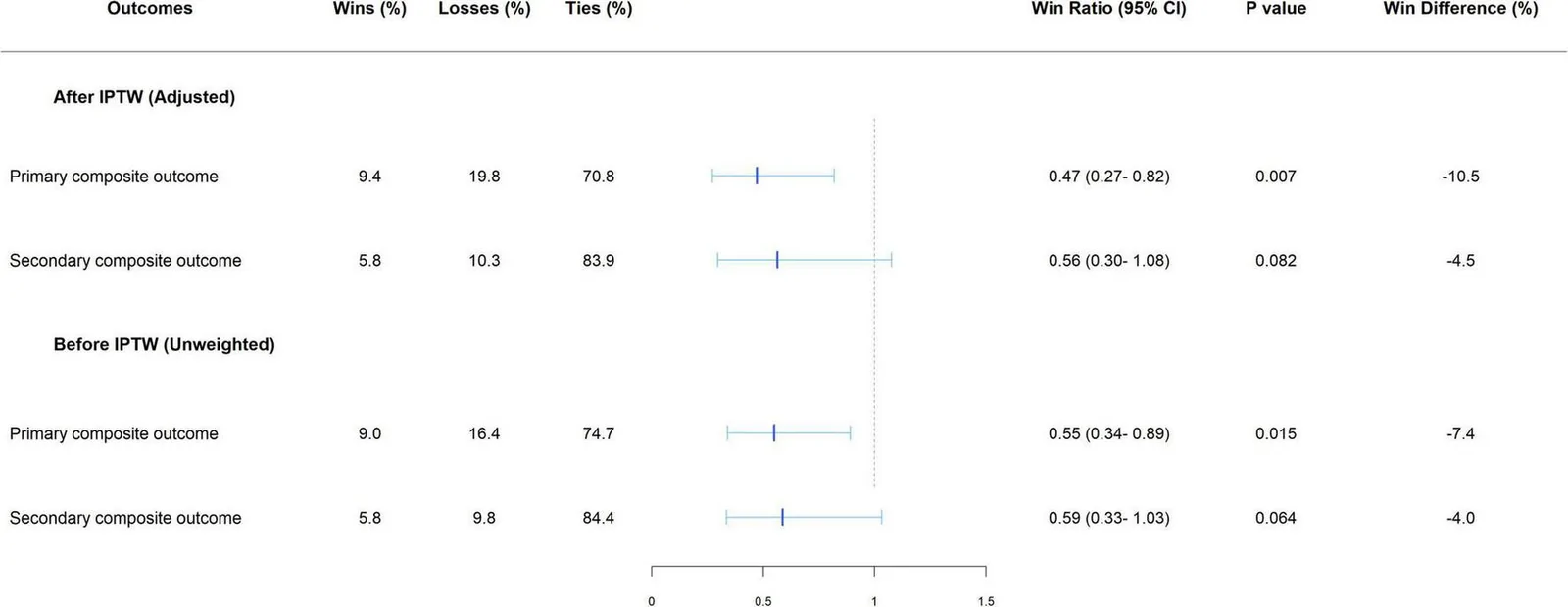
Forest plot of the IPTW-adjusted win ratio analysis. The figure illustrates the Win Ratios and 95% confidence intervals comparing the RD-ABV group with the non-RD-ABV group in the weighted cohort. The primary composite outcome was analyzed using a hierarchical approach consisting of mRS outcome (mRS 3–6), cerebral infarction, cerebral hemorrhage, and reoperation. The secondary composite outcome was analyzed using a hierarchical approach consisting of cerebral infarction, cerebral hemorrhage and reoperation. IPTW, inverse probability of treatment weighting; RD-ABV, residual dissection in arch branch vessels; mRS, modified Rankin Scale; CI, confidence interval.

Figure 2 displays the win ratio results for the primary and secondary composite outcomes before and after IPTW. After IPTW, the win ratios for the primary and secondary composite outcomes were 0.47 (95% CI: 0.27–0.82; *P* = 0.007) and 0.56 (95% CI: 0.30–1.08; *P* = 0.082), respectively. Before IPTW, the win ratios for the primary and secondary composite outcomes were 0.55 (95% CI: 0.34–0.89; *P* = 0.015) and 0.59 (95% CI: 0.33–1.03; *P* = 0.064), respectively. The distribution of specific reoperation types during follow-up was shown in Supplementary Figure 1.

### 3.3 Hierarchical decomposition of the primary composite outcome

**FIGURE 3:**
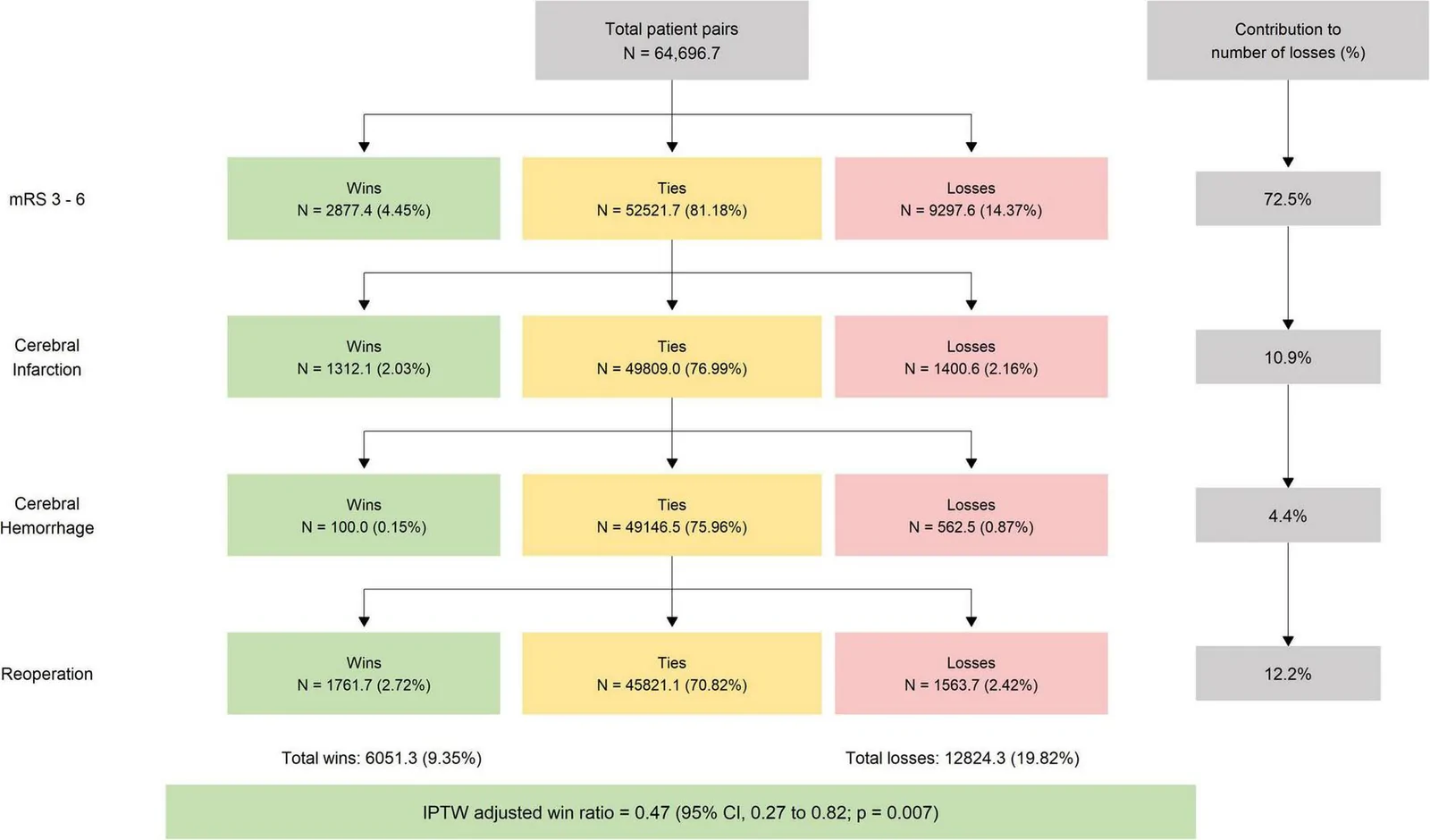
IPTW-adjusted Win Ratio for the hierarchical composite outcome analysis of mRS 3–6, CI, CH or reoperation in RD-ABV versus non-RD-ABV group.

A hierarchical outcome analysis was conducted using the IPTW approach, as described in the Methods section. All patients in the RD-ABV group (*n* = 156) were paired with all patients in the non-RD-ABV group (*n* = 408), resulting in 63,648 physical matched pairs and 64,696.7 weighted patient pairs. In the weighted win ratio analysis, the RD-ABV group had 6,051.3 wins (9.35%), 12,824.3 losses (19.82%), and 45,821.1 ties (70.82%). The overall win ratio was 0.47 (95% CI: 0.27–0.82; *P* = 0.007), indicating a lower likelihood of a favorable hierarchical outcome in the RD-ABV group. The largest contributor to losses was mRS 3–6, which accounted for 72.5% of all losses (9,297.6 losses vs. 2,877.4 wins). Cerebral infarction accounted for 10.9% of losses (1,400.6 losses vs. 1,312.1 wins), cerebral hemorrhage for 4.4% (562.5 losses vs. 100.0 wins), and reoperation for 12.2% (1,563.7 losses vs. 1,761.7 wins). The comprehensive breakdown of the hierarchical analysis is illustrated in Figure 3.

### 3.4 Dynamic evolution of win ratio and win proportions over follow-up

**FIGURE 4:**
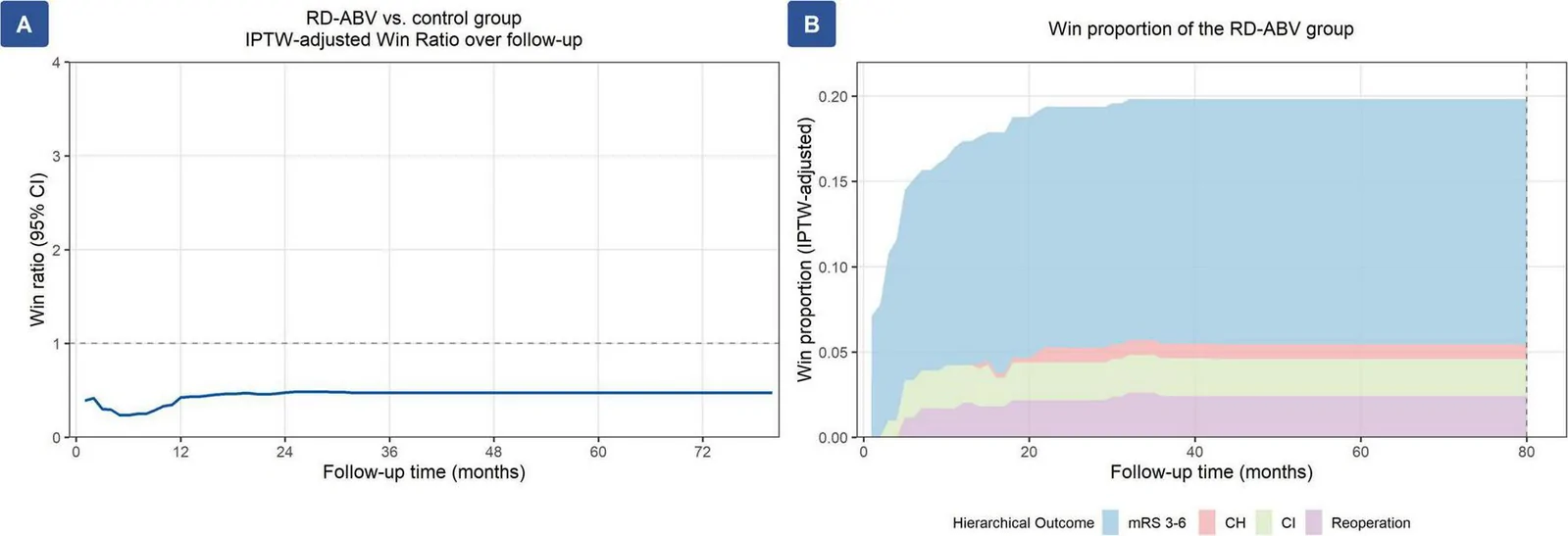
Changes over the course of follow-up of: the IPTW-adjusted Win Ratio with 95% confidence interval in RD-ABV versus non-RD-ABV group **(A)** and win proportion of the RD-ABV group **(B).**

To evaluate the temporal stability of the observed association, dynamic changes in the IPTW-adjusted win ratio were analyzed throughout follow-up. The win ratio for the hierarchical time-to-event outcomes decreased during the early follow-up period, reached its lowest value at approximately 6 months, then gradually increased and stabilized by approximately 24 to 36 months (Figure 4A). The curve remained consistently below the 1.0 threshold throughout the study duration. This pattern supports an association between RD-ABV and a lower probability of a favorable hierarchical outcome, but it should not be interpreted as evidence of causality.

Analysis of the hierarchical outcome contributions (Figure 4B) further clarified the specific drivers of losses in the RD-ABV group. The cumulative loss proportion rose rapidly during early follow-up and then reached a plateau. mRS 3–6 was the dominant contributor throughout follow-up, whereas cerebral infarction, cerebral hemorrhage, and reoperation contributed smaller proportions to the total losses.

### 3.5 Sensitivity analysis

The median follow-up time for the primary outcome was 18.0 months (IQR: 6.0–29.3) in the RD-ABV group and 16.0 months (IQR: 10.8–27.0) in the non-RD-ABV group. IPTW-weighted Kaplan-Meier curves for the composite outcomes and their individual components were presented in Supplementary Figure 2. In this sensitivity analysis, IPTW-weighted Kaplan-Meier curves were used to compare event-free survival between groups. Raw event-free counts were reported for descriptive purposes, whereas survival probabilities and P values were estimated in the IPTW-weighted cohort. The RD-ABV group had a lower IPTW-weighted event-free probability for the primary outcome, with raw event-free counts of 123/156 (78.8%) versus 355/408 (87.0%) in the non-RD-ABV group (weighted log-rank *P* = 0.014). Similar findings were observed for mRS 3–6 [133/156 (85.3%) vs. 387/408 (94.9%); weighted log-rank *P* < 0.001] and CH [151/156 (96.8%) vs. 406/408 (99.5%); weighted log-rank *P* = 0.034]. In contrast, the secondary composite outcome [134/156 (85.9%) vs. 373/408 (91.4%); weighted log-rank *P* = 0.058], CI [145/156 (92.9%) vs. 393/408 (96.3%); weighted log-rank *P* = 0.132], and reoperation [144/156 (92.3%) vs. 389/408 (95.3%); weighted log-rank *P* = 0.091] did not reach statistical significance.

After IPTW adjustment, Cox proportional hazards regression analysis was performed to evaluate factors associated with the primary composite outcome (Supplementary Figure 3). The full weighted multivariable Cox model is provided in Supplementary Table 2, and the corresponding univariable Cox screening used for covariate selection is shown in Supplementary Table 3. In the weighted multivariable Cox model, RD-ABV was associated with a higher hazard of the primary composite outcome after adjustment for measured covariates (HR = 1.92; 95% CI: 1.23–3.00; *P* = 0.004). Advanced age was also associated with a higher hazard of the primary composite outcome (HR = 1.02; 95% CI: 1.00–1.05; *P* = 0.025). Albumin showed only a weak association in the weighted multivariable model (HR = 1.01; 95% CI: 1.00–1.03; *P* = 0.037), and this exploratory finding should be interpreted cautiously. The remaining selected covariates did not reach statistical significance.

Because WBC and neutrophil count were highly correlated, a reduced propensity score sensitivity analysis excluding WBC was performed. In the original propensity score model, the Pearson correlation between WBC and neutrophil count was 0.983, and the VIF values for WBC and neutrophil count were both > 100, indicating substantial collinearity. After excluding WBC, the weight distribution became more stable (maximum stabilized weight, 4.35 vs. 6.98; overall ESS, 521.1 vs. 497.8), and covariate balance was similar or slightly improved (mean post-IPTW SMD, 0.030 vs. 0.040; one covariate vs. two covariates with SMD > 0.10). Outcome estimates were consistent with the primary analysis, including the primary win ratio (0.49; 95% CI: 0.29–0.82; *P* = 0.007) and the secondary win ratio (0.56; 95% CI: 0.31–1.02; *P* = 0.056). Details are shown in Supplementary Table 9.

## 4 Discussion

This retrospective cohort study, which included 156 patients with RD-ABV and 408 non-RD-ABV patients, showed that RD-ABV was associated with worse mid-term post-discharge functional recovery among selected survivors after total aortic arch replacement for type A aortic dissection. The statistically significant primary composite outcome was mainly driven by mRS 3–6, whereas the secondary composite outcome excluding mRS did not reach statistical significance. Therefore, the present findings should be interpreted as evidence of an association with functional recovery, rather than as proof that RD-ABV directly causes hard cerebrovascular events or reoperation. These results support closer postoperative attention to patients with residual distal branch vessel dissection and provide a basis for future prospective studies.

The findings of this study, which suggest an association between RD-ABV and worse long-term functional recovery, are consistent with prior evidence and extend the current literature. Previous research has shown that malperfusion of the supra-aortic vessels during the acute phase of type A aortic dissection is associated with in-hospital mortality and neurological morbidity (21). For instance, registry data have indicated that preoperative cerebral malperfusion is associated with worse surgical outcomes (21). Our study shifted the focus to the chronic postoperative period and addressed a notable gap in the current evidence. The results suggest that, even after successful surgical repair of type A aortic dissection, persistent dissection within the aortic arch branches may be linked to poorer neurological and functional recovery. To provide further clinical context, we performed a descriptive analysis of the 31 reoperation events. We observed that cerebrovascular interventions (neuro-interventions, carotid endarterectomy, carotid artery stenting) accounted for 38.7% of all reoperations, while coronary interventions constituted another 29.0%. Because formal correlation analysis was not performed due to sample size, these findings should be viewed as descriptive and hypothesis-generating.

Furthermore, the association between a patent false lumen in the downstream aorta and long-term aortic expansion, reintervention, and mortality has been well-established in studies of both acute and chronic aortic dissections (14, 22). Tsai et al. reported that partial thrombosis of the false lumen was associated with late mortality in patients with type B dissection (14). Our results are consistent with the broader concept that persistent false lumen flow may be clinically relevant after aortic dissection repair. However, in the present cohort, the main statistical signal was related to poor functional recovery. Although cerebral hemorrhage was more frequent in the RD-ABV group, the absolute number of events was small, and this finding should be interpreted cautiously. The incidence of cerebral infarction and reoperation showed increasing trends but did not reach statistical significance.

It is noteworthy that some earlier reports have found that the mere presence of residual dissection may not invariably lead to poor survival and suggested a more conservative approach in the short term (23). Our findings do not contradict this view because the secondary composite outcome excluding mRS was not statistically significant. Instead, our results suggest that mRS may capture a broader spectrum of functional impairment after discharge, which may be more sensitive to the neurological sequelae associated with RD-ABV than mortality or reoperation alone.

Taken together, this study highlights the potential clinical relevance of RD-ABV after repair of type A aortic dissection. It extends the existing focus on acute malperfusion and early outcomes by suggesting that residual branch vessel dissection may be associated with worse post-discharge functional recovery. These findings refine our understanding of the chronic course after repair, but prospective validation is required before RD-ABV can be used to guide targeted therapeutic strategies.

Furthermore, we examined the potential confounding influence of long-term postoperative medications on clinical outcomes (detailed in Supplementary Table 1). Our analysis showed no significant differences between the RD-ABV and non-RD-ABV groups regarding the use of antiplatelet or anticoagulant agents, which are traditional drivers of hemorrhagic complications (24). Interestingly, the utilization of calcium channel blockers was significantly higher in the non-RD-ABV group, a finding that parallels the higher baseline prevalence of hypertension in non-RD-ABV patients. This observation suggests that the association between RD-ABV and poorer functional recovery is unlikely to be explained solely by differences in antiplatelet, anticoagulant, or antihypertensive medication use. Nevertheless, residual confounding cannot be excluded in this retrospective analysis.

This study had several limitations. First, because of its retrospective single-center design, selection bias, attrition bias, and residual confounding remain possible despite IPTW adjustment. Patients with in-hospital postoperative CI, CH, or death and 89 otherwise eligible patients with no post-discharge follow-up were excluded; thus, the findings apply only to selected post-discharge survivors with available follow-up. Key operative and anatomical factors, including dissection acuity, DeBakey classification, preoperative malperfusion, cannulation strategy, circulatory arrest time, cerebral perfusion strategy, frozen elephant trunk use, branch reconstruction technique, and surgeon or time-period effects, were not available with sufficient completeness or standardized definitions. Complete-case adjustment would have reduced the analytic sample and introduced additional selection bias; therefore, these factors were not included in the IPTW or multivariable models.

Second, RD-ABV was primarily analyzed as a binary exposure. Supplementary Table 8 describes involved branches and branch counts, but limited subgroup sizes and events precluded stable analyses by anatomical pattern. Routine postoperative CTA assessed the arch and proximal branch vessels but not dedicated head and neck CTA findings; false lumen patency, true lumen narrowing, thrombosis status, distal carotid or vertebral extension, and malperfusion signs were not systematically annotated. Finally, mRS assessment from records or structured telephone follow-up may introduce information bias despite assessor training and blinding, and cerebral hemorrhage events were few.

In the absence of established clinical guidelines for the management of RD-ABV, the findings of the present study outline several promising directions for future research. First, large-scale prospective multicenter studies are needed to validate the generalizability of our results. Subsequent efforts should focus on analyzing the specific association between residual dissection in different branch vessels and clinical outcomes, and on incorporating advanced imaging technologies, such as computational fluid dynamics, to elucidate underlying mechanisms from both hemodynamic and anatomical perspectives. Future studies may evaluate whether selected high-risk patients benefit from additional branch vessel intervention, but prophylactic branch vessel stent implantation should be regarded only as a hypothesis for future research. Dedicated research is essential to advance personalized treatment strategies and improve long-term outcomes in this patient population.

## 5 Conclusion

In this retrospective cohort study of selected patients discharged after total arch replacement, residual dissection in the aortic arch branch vessels was associated with worse mid-term functional recovery. The primary composite outcome was mainly driven by mRS 3–6, while the secondary composite outcome excluding mRS did not reach statistical significance. These findings support the clinical relevance of RD-ABV as a potential marker for postoperative risk stratification and closer follow-up, but they should be regarded as hypothesis-generating. Further prospective studies with detailed anatomical assessment are needed to validate these findings and determine whether any targeted management strategy can improve long-term outcomes.

## Data Availability

The raw data supporting the conclusions of this article will be made available by the authors, without undue reservation.
