# Supplementary material for: Clinical outcomes of residual branch vessel dissection following type A aortic dissection repair
Source: Front Med (Lausanne). 2026 Aug 26;13:1913510. doi: 10.3389/fmed.2026.1913510 (PMC13553776; doi:10.3389/fmed.2026.1913510)
Supplement: Supplementary file 1 [file Data_Sheet_1.docx]

**Figure S1:** Distribution of specific reoperation types during the follow-up period. The donut chart illustrates the breakdown of surgical and interventional procedures performed on the 31 patients who required reoperation. The segments display the absolute number of patients for each procedure, with the corresponding percentage of the total reoperations (N=31) shown in parentheses.

PCI, percutaneous coronary intervention; AV, aortic valve; CEA, carotid endarterectomy; CAS, carotid artery stenting; CABG, coronary artery bypass grafting; ET, elephant trunk; MVR, mitral valve replacement.

**Table S1: Long-term medication after discharge of two groups.**

| **Long-term medication after discharge (%)** | **RD-ABV group (n=156)** | **Control group**  **(n=408)** | ***P* value** |
| --- | --- | --- | --- |
| β-blocker | 102 (65.4) | 261 (64.0) | 0.829 |
| Diuretics | 114 (73.1) | 293 (71.8) | 0.846 |
| Sacubitril/Valsartan | 16 (10.3) | 43 (10.5) | >0.999 |
| ACEI | 5 (3.2) | 13 (3.2) | >0.999 |
| CCB | 52 (33.3) | 195 (47.8) | 0.003 |
| Anticoagulants | 29 (18.6) | 69 (16.9) | 0.729 |
| Antiplatelet agents | 11 (7.1) | 41 (10.0) | 0.348 |
| Lipid-lowering drug | 52 (33.3) | 128 (31.4) | 0.729 |

ACEI, angiotensin-converting enzyme inhibitors; CCB, calcium channel blockers.

Figure S2: IPTW-weighted Kaplan-Meier event-free survival curves. The panels display event-free survival probabilities in the weighted cohort for: (A) primary composite outcome; (B) secondary composite outcome; (C) mRS outcome; (D) cerebral infarction; (E) cerebral hemorrhage; and (F) reoperation. Event counts are reported as raw numbers, whereas survival probabilities and P values were estimated in the IPTW-weighted cohort. IPTW, inverse probability of treatment weighting; mRS, modified Rankin Scale.

**Figure S3:** Multivariable Cox proportional hazards regression analysis for the primary composite outcome in the IPTW-weighted cohort. HR, hazard ratio; CI, confidence interval; IPTW, inverse probability of treatment weighting. Covariates shown in this model were selected from univariable screening using a P<0.10 threshold and modeled together with RD-ABV status.

**Table S2:** Multivariable Cox proportional hazards regression analysis for the primary composite outcome in the IPTW-weighted cohort.

| **Variable** | **HR** | **95% CI** | **P value** |
| --- | --- | --- | --- |
| RD-ABV | 1.92 | 1.23-3.00 | 0.004 |
| Diabetes | 1.65 | 0.60-4.54 | 0.333 |
| Age | 1.02 | 1.00-1.05 | 0.025 |
| BUN | 1.04 | 0.97-1.13 | 0.276 |
| Albumin | 1.01 | 1.00-1.03 | 0.037 |
| TBIL | 1.02 | 1.00-1.04 | 0.052 |
| DBIL | 1.02 | 0.99-1.06 | 0.227 |
| AST | 1.00 | 1.00-1.00 | 0.261 |
| ALT | 1.00 | 0.99-1.01 | 0.837 |

Variables with P<0.10 in univariable Cox screening, together with RD-ABV status, were included in the multivariable model. Stabilized IPTW weights and robust variance estimation were used. HR, hazard ratio; CI, confidence interval; RD-ABV, residual dissection in aortic arch branch vessels; BUN, blood urea nitrogen.

**Table S3:** Univariable Cox proportional hazards screening for the primary composite outcome in the IPTW-weighted cohort.

| **Variable** | **HR** | **95% CI** | **P value** | **Entered model** |
| --- | --- | --- | --- | --- |
| Male sex | 0.82 | 0.43-1.57 | 0.556 | No |
| Smoking | 0.85 | 0.52-1.38 | 0.507 | No |
| Drinking | 0.97 | 0.56-1.66 | 0.906 | No |
| Hypertension | 1.03 | 0.62-1.70 | 0.919 | No |
| Coronary heart disease | 1.17 | 0.59-2.32 | 0.660 | No |
| Heart valve disease | 1.66 | 0.74-3.69 | 0.216 | No |
| Diabetes | 2.27 | 0.92-5.57 | 0.075 | Yes |
| Age | 1.02 | 1.00-1.05 | 0.050 | Yes |
| RBC | 0.98 | 0.68-1.40 | 0.911 | No |
| WBC | 1.00 | 0.95-1.06 | 0.890 | No |
| Neutrophils | 1.00 | 0.95-1.06 | 0.936 | No |
| Lymphocytes | 0.83 | 0.47-1.46 | 0.514 | No |
| Platelet count | 1.00 | 0.99-1.00 | 0.334 | No |
| D-dimer | 1.00 | 0.99-1.01 | 0.975 | No |
| Fibrinogen | 1.05 | 0.92-1.20 | 0.441 | No |
| TT | 1.00 | 0.99-1.01 | 0.933 | No |
| APTT | 0.98 | 0.94-1.03 | 0.477 | No |
| PT | 0.98 | 0.91-1.06 | 0.603 | No |
| INR | 1.14 | 0.87-1.48 | 0.348 | No |
| Creatinine | 1.00 | 1.00-1.00 | 0.122 | No |
| BUN | 1.08 | 1.01-1.14 | 0.016 | Yes |
| Albumin | 1.01 | 1.00-1.02 | 0.071 | Yes |
| TBIL | 1.03 | 1.00-1.05 | 0.036 | Yes |
| DBIL | 1.06 | 1.00-1.11 | 0.032 | Yes |
| AST | 1.00 | 1.00-1.00 | 0.015 | Yes |
| ALT | 1.00 | 1.00-1.01 | 0.048 | Yes |

Variables with P<0.10 were entered into the multivariable Cox model together with RD-ABV status. RD-ABV status was retained as the exposure of interest regardless of univariable screening. Stabilized IPTW weights and robust variance estimation were used. HR, hazard ratio; CI, confidence interval; BUN, blood urea nitrogen; INR, international normalized ratio.

**Propensity score and IPTW details**

The propensity score was estimated using logistic regression with RD-ABV status as the dependent variable. Only covariates shown in the original Table 1 were included in the propensity score model. Stabilized inverse probability of treatment weights were used for the primary weighted analyses. Covariate balance was assessed using absolute standardized mean differences, with values below 0.10 considered acceptable. Weighted Cox models used robust variance estimation. Because WBC, neutrophil count, and lymphocyte count were highly correlated, coefficients for these variables in the propensity score model were interpreted as model-based balancing parameters rather than independent etiological predictors.

**Table S4:** Propensity score and IPTW analysis specification.

| **Item** | **Description** |
| --- | --- |
| Treatment/exposure definition | group=1, residual dissection in aortic arch branch vessels (RD-ABV) |
| Control definition | group=0, non-RD-ABV |
| Propensity score model | Logistic regression using only covariates shown in the original Table 1: sex_male, smoking, drinking, HTN, CHD, HVD, diabetes, age, RBC, WBC, NEU, Lymph, platelet, D_dimer, fibrinogen, TT, APTT, PT, PTINT, Cr, Urea, albumin, TBIL, DBIL, AST, ALT |
| IPTW formula | For RD-ABV: P(T=1)/PS. For non-RD-ABV: P(T=0)/(1-PS). |
| Stabilized weights | Yes, stabilized IPTW was used for the primary weighted analyses. |
| Weight truncation primary analysis | No truncation was applied in the primary analysis because weights were not extreme. |
| Truncated weights sensitivity | 1st and 99th percentile limits were 0.483 and 2.083; truncated weights were generated for diagnostics only. |
| Balance criterion | Absolute standardized mean difference <0.1 was considered adequate balance. |
| mRS endpoint definition | mRS 3-6 was defined as mRS >=3. mRS 6 was included as death during follow-up. |
| Primary composite endpoint | mRS 3-6, cerebral infarction, cerebral hemorrhage, or cardiovascular/cerebrovascular reoperation. |
| Secondary composite endpoint | Cerebral infarction, cerebral hemorrhage, or cardiovascular/cerebrovascular reoperation. |

Abbreviations: IPTW, inverse probability of treatment weighting; PS, propensity score; RD-ABV, residual dissection in aortic arch branch vessels.

**Table S5:** Full propensity score model.

| **Covariate** | **Logit coefficient** | **SE** | **OR** | **95% CI** | **P value** |
| --- | --- | --- | --- | --- | --- |
| Intercept | 3.283 | 1.561 | 26.654 | 1.251-567.750 | 0.035 |
| Gender-male (n, %) | 0.121 | 0.312 | 1.129 | 0.612-2.082 | 0.698 |
| Smoking (n, %) | -0.017 | 0.256 | 0.983 | 0.596-1.624 | 0.948 |
| Alcohol use (n, %) | -0.064 | 0.252 | 0.938 | 0.572-1.536 | 0.799 |
| Hypertension | -0.616 | 0.213 | 0.540 | 0.355-0.820 | 0.004 |
| CHD | 0.290 | 0.323 | 1.337 | 0.710-2.516 | 0.368 |
| VHD | 0.085 | 0.544 | 1.089 | 0.375-3.161 | 0.875 |
| Diabetes | 0.736 | 0.604 | 2.087 | 0.639-6.820 | 0.223 |
| Age (years) | -0.009 | 0.010 | 0.991 | 0.972-1.010 | 0.350 |
| RBC (10^12/L) | -0.304 | 0.214 | 0.738 | 0.486-1.122 | 0.155 |
| WBC (10^9/L) | 1.097 | 0.421 | 2.995 | 1.313-6.832 | 0.009 |
| NEU (10^9/L) | -1.126 | 0.434 | 0.324 | 0.139-0.759 | 0.009 |
| LYMPH (10^9/L) | -2.067 | 0.610 | 0.127 | 0.038-0.419 | <0.001 |
| Platelet (10^9/L) | 0.001 | 0.002 | 1.001 | 0.997-1.004 | 0.754 |
| D-dimer (mg/L) | 0.004 | 0.004 | 1.004 | 0.996-1.012 | 0.300 |
| Fibrinogen (g/L) | -0.099 | 0.091 | 0.906 | 0.757-1.084 | 0.281 |
| TT (s) | 0.014 | 0.009 | 1.014 | 0.997-1.032 | 0.117 |
| APTT (s) | -0.002 | 0.018 | 0.998 | 0.964-1.034 | 0.913 |
| PT (s) | -0.122 | 0.070 | 0.885 | 0.771-1.016 | 0.083 |
| INR | 0.327 | 0.475 | 1.387 | 0.546-3.520 | 0.492 |
| Cr (umol/L) | -0.001 | 0.002 | 0.999 | 0.995-1.004 | 0.793 |
| BUN (mmol/L) | 0.017 | 0.051 | 1.018 | 0.920-1.126 | 0.735 |
| Albumin (g/L) | -0.002 | 0.007 | 0.998 | 0.983-1.012 | 0.740 |
| TBIL (umol/L) | -0.010 | 0.010 | 0.990 | 0.970-1.010 | 0.322 |
| DBIL (umol/L) | -0.006 | 0.028 | 0.994 | 0.942-1.049 | 0.828 |
| AST (U/L) | -0.004 | 0.004 | 0.996 | 0.989-1.003 | 0.284 |
| ALT (U/L) | -0.000 | 0.005 | 1.000 | 0.989-1.010 | 0.987 |

All covariates listed in the original Table 1 were included in the propensity score model. ORs describe the odds of RD-ABV. WBC, NEU, and LYMPH showed substantial collinearity in this model; therefore, a reduced propensity score sensitivity analysis excluding WBC was performed and is summarized in Table S9.

**Table S6:** Weight distribution, effective sample size, and propensity score overlap.

| **Group** | **N** | **Mean stabilized weight** | **Weight range** | **ESS** | **PS range** | **Outside common support** |
| --- | --- | --- | --- | --- | --- | --- |
| RD-ABV | 156 | 1.019 | 0.404-6.982 | 112.2 | 0.040-0.684 | 6 |
| Non-RD-ABV | 408 | 0.998 | 0.723-2.037 | 396.1 | 0.000-0.645 | 2 |
| Overall | 564 |  |  | 497.8 |  |  |

Common support was defined by the overlapping range of propensity scores between the RD-ABV and non-RD-ABV groups. ESS, effective sample size.

**Table S7:** Covariate balance before and after IPTW.

| **Covariate** | **Type** | **SMD before IPTW** | **SMD after IPTW** | **Balanced after IPTW** |
| --- | --- | --- | --- | --- |
| Gender-male (n, %) | categorical | 0.055 | 0.048 | Yes |
| Age (years) | continuous | 0.007 | 0.029 | Yes |
| Smoking (n, %) | categorical | 0.033 | 0.042 | Yes |
| Alcohol use (n, %) | categorical | 0.041 | 0.047 | Yes |
| Hypertension | categorical | 0.279 | 0.031 | Yes |
| CHD | categorical | 0.048 | 0.017 | Yes |
| VHD | categorical | 0.036 | 0.030 | Yes |
| Diabetes | categorical | 0.079 | 0.004 | Yes |
| RBC (10^12/L) | continuous | 0.168 | 0.052 | Yes |
| WBC (10^9/L) | continuous | 0.032 | 0.012 | Yes |
| NEU (10^9/L) | continuous | 0.008 | 0.014 | Yes |
| LYMPH (10^9/L) | continuous | 0.279 | 0.019 | Yes |
| Platelet (10^9/L) | continuous | 0.087 | 0.003 | Yes |
| D-dimer (mg/L) | continuous | 0.127 | 0.037 | Yes |
| Fibrinogen (g/L) | continuous | 0.087 | 0.064 | Yes |
| TT (s) | continuous | 0.015 | 0.038 | Yes |
| APTT (s) | continuous | 0.028 | 0.001 | Yes |
| PT (s) | continuous | 0.041 | 0.065 | Yes |
| INR | continuous | 0.008 | 0.033 | Yes |
| Cr (umol/L) | continuous | 0.019 | 0.005 | Yes |
| BUN (mmol/L) | continuous | 0.081 | 0.039 | Yes |
| Albumin (g/L) | continuous | 0.024 | 0.009 | Yes |
| TBIL (umol/L) | continuous | 0.110 | 0.151 | No |
| DBIL (umol/L) | continuous | 0.065 | 0.176 | No |
| AST (U/L) | continuous | 0.152 | 0.045 | Yes |
| ALT (U/L) | continuous | 0.164 | 0.017 | Yes |

Absolute standardized mean differences below 0.10 were considered acceptable. Values are reported as absolute SMDs.

**Figure S4:** Propensity score overlap after model estimation. Density plots show the distribution of estimated propensity scores in the RD-ABV and non-RD-ABV groups.

**Figure S5:** Covariate balance before and after IPTW. The love plot shows absolute standardized mean differences for all covariates included in the propensity score model before and after weighting. The dashed line indicates the 0.10 balance threshold.

**Figure S6:** Distribution of stabilized IPTW weights. Boxplots summarize the stabilized inverse probability of treatment weights in the RD-ABV and non-RD-ABV groups.

**Table S8:** Anatomical distribution of residual dissection in aortic arch branch vessels among patients with RD-ABV.

| **Anatomical feature** | **n (% of RD-ABV patients, n=156)** |
| --- | --- |
| **Specific involved branch vessel** |  |
| Brachiocephalic trunk | 105 (67.3) |
| Left common carotid artery | 46 (29.5) |
| Left subclavian artery | 45 (28.8) |
| **Number of involved branch vessels** |  |
| One branch | 124 (79.5) |
| Two branches | 24 (15.4) |
| Three branches | 8 (5.1) |
| **Anatomical involvement pattern** |  |
| Brachiocephalic trunk only | 77 (49.4) |
| Left common carotid artery only | 22 (14.1) |
| Left subclavian artery only | 25 (16.0) |
| Brachiocephalic trunk + left common carotid artery | 12 (7.7) |
| Brachiocephalic trunk + left subclavian artery | 8 (5.1) |
| Left common carotid artery + left subclavian artery | 4 (2.6) |
| All three arch branches | 8 (5.1) |

Values are n (% of patients with RD-ABV). Specific involved branch vessel categories are not mutually exclusive, whereas anatomical involvement patterns are mutually exclusive. Formal outcome analysis by anatomical involvement pattern was not performed because subgroup sizes and event counts were limited. RD-ABV, residual dissection in aortic arch branch vessels.

**Table S9**: Sensitivity propensity score analysis excluding WBC because of collinearity among leukocyte variables.

| **Domain** | **Measure** | **Original PS model (WBC included)** | **Reduced PS model (WBC excluded)** |
| --- | --- | --- | --- |
| Collinearity | Leukocyte variables in PS model | WBC, NEU, and LYMPH | NEU and LYMPH |
| Collinearity | Pearson correlation between WBC and NEU | 0.983 | Not applicable |
| Collinearity | VIF for WBC / NEU / LYMPH | 103.37 / 103.27 / 3.74 | WBC excluded |
| PS coefficients | WBC OR (95% CI), P value | 2.99 (1.31-6.83), P=0.009 | Excluded |
| PS coefficients | NEU OR (95% CI), P value | 0.32 (0.14-0.76), P=0.009 | 1.00 (0.94-1.06), P=0.998 |
| PS coefficients | LYMPH OR (95% CI), P value | 0.13 (0.04-0.42), P<0.001 | 0.53 (0.32-0.87), P=0.012 |
| Weights | Maximum stabilized IPTW | 6.98 | 4.35 |
| Weights | Overall ESS | 497.8 | 521.1 |
| Weights | RD-ABV ESS | 112.2 | 126.7 |
| Balance | Mean SMD after IPTW | 0.040 | 0.030 |
| Balance | Covariates with SMD >0.10 after IPTW | 2 (TBIL, DBIL) | 1 (DBIL) |
| Leukocyte balance | WBC / NEU / LYMPH SMD after IPTW | 0.012 / 0.014 / 0.019 | 0.009 / 0.031 / 0.007 |
| Outcome | Primary win ratio (95% CI), P value | 0.47 (0.27-0.82), P=0.007 | 0.49 (0.29-0.82), P=0.007 |
| Outcome | Secondary win ratio (95% CI), P value | 0.56 (0.30-1.08), P=0.082 | 0.56 (0.31-1.02), P=0.056 |
| Outcome | IPTW-weighted marginal Cox HR for primary composite outcome | 1.94 (1.15-3.27), P=0.014 | 1.89 (1.17-3.05), P=0.009 |

The Cox models in Table S9 were marginal IPTW-weighted models including RD-ABV status only and were used to compare propensity score weighting strategies. Table S2 reports the IPTW-weighted multivariable Cox model additionally adjusted for selected covariates.

Abbreviations: BUN, blood urea nitrogen; CI, confidence interval; DBIL, direct bilirubin; ESS, effective sample size; HR, hazard ratio; INR, international normalized ratio; IPTW, inverse probability of treatment weighting; LYMPH, lymphocyte count; NEU, neutrophil count; OR, odds ratio; PS, propensity score; RD-ABV, residual dissection in aortic arch branch vessels; SMD, standardized mean difference; TBIL, total bilirubin; VIF, variance inflation factor; WBC, white blood cell count; WR, win ratio.
